# Understanding and monitoring the consequences of human impacts on intraspecific variation

**DOI:** 10.1111/eva.12436

**Published:** 2016-11-22

**Authors:** Makiko Mimura, Tetsukazu Yahara, Daniel P. Faith, Ella Vázquez‐Domínguez, Robert I. Colautti, Hitoshi Araki, Firouzeh Javadi, Juan Núñez‐Farfán, Akira S. Mori, Shiliang Zhou, Peter M. Hollingsworth, Linda E. Neaves, Yuya Fukano, Gideon F. Smith, Yo‐Ichiro Sato, Hidenori Tachida, Andrew P. Hendry

**Affiliations:** ^1^ Department of Bioenvironmental SystemsTamagawa UniversityTokyoJapan; ^2^ Department of Biology and Institute of Decision Science for a Sustainable SocietyKyushu UniversityFukuokaJapan; ^3^ The Australian Museum Research InstituteThe Australian MuseumSydneyNSWAustralia; ^4^ Instituto de EcologíaUniversidad Nacional Autónoma de MéxicoMéxicoMéxico; ^5^ Biology DepartmentQueen's UniversityKingstonONCanada; ^6^ Research Faculty of AgricultureHokkaido UniversitySapporoHokkaidoJapan; ^7^ Graduate School of Environment and Information SciencesYokohama National UniversityYokohamaJapan; ^8^ State Key Laboratory of Systematic and Evolutionary BotanyInstitute of BotanyChinese Academy of SciencesBeijingChina; ^9^ Royal Botanic Garden EdinburghEdinburghUK; ^10^ Australian Centre for Wildlife Genomics, Australian Museum Research InstituteAustralian MuseumSydneyNSWAustralia; ^11^ Department of BotanyNelson Mandela Metropolitan UniversityPort ElizabethSouth Africa; ^12^ Departamento de Ciências da VidaCentre for Functional EcologyUniversidade de CoimbraCoimbraPortugal; ^13^ National Institute for HumanitiesTokyoJapan; ^14^ Redpath Museum and Department of BiologyMcGill UniversityMontrealQuebecCanada

**Keywords:** ecosystem function and services, functional variation, genetic variation, neutral variation, non‐neutral variation

## Abstract

Intraspecific variation is a major component of biodiversity, yet it has received relatively little attention from governmental and nongovernmental organizations, especially with regard to conservation plans and the management of wild species. This omission is ill‐advised because phenotypic and genetic variations within and among populations can have dramatic effects on ecological and evolutionary processes, including responses to environmental change, the maintenance of species diversity, and ecological stability and resilience. At the same time, environmental changes associated with many human activities, such as land use and climate change, have dramatic and often negative impacts on intraspecific variation. We argue for the need for local, regional, and global programs to monitor intraspecific genetic variation. We suggest that such monitoring should include two main strategies: (i) intensive monitoring of multiple types of genetic variation in selected species and (ii) broad‐brush modeling for representative species for predicting changes in variation as a function of changes in population size and range extent. Overall, we call for collaborative efforts to initiate the urgently needed monitoring of intraspecific variation.

## 1 Introduction

The future of the Earth system will be heavily influenced by human‐induced environmental changes that have detrimental effects on biodiversity. The consequent loss of diversity impacts not only local ecosystems and the services they provide (Cardinale et al., 2012; Díaz, Fargione, Chapin, & Tilman, 2006), but also biodiversity on regional and global scales (Faith et al., 2010). Under the Convention on Biological Diversity (CBD), biodiversity is considered to encompass variation at all levels, such as within and among ecosystems, communities, species, and populations. However, to date, most discussions and efforts that considered the impacts of human‐induced environmental change focused on ecosystems and communities. Although some programs, such as the US Endangered Species Act (ESA) and Canadian Species at Risk Act (SARA), have long cited the importance of intraspecific variation for protecting endangered species, intraspecific variation is typically overlooked (Hoban et al., 2013; Laikre, 2010), especially for nonendangered but possibly ecologically important species. Here, we argue that ignoring intraspecific variation in management decisions can lead to irreversible consequences for biodiversity and the services and benefits it provides. In particular, we will argue that human activities are dramatically changing the structure of neutral and functional variations in natural populations that are critical for species persistence, community structure, and ecosystem services and hence the integration of strategies to monitor this variation is urgently required.

In this review, “intraspecific variation” refers to all forms of variation within a species, both within and among populations, including variations in phenotypes and genomes. “Genetic variation” refers to all forms of genetic variation within a species, including neutral and functional sequence variations and variation in gene expression. The term “molecular genetic diversity’” is used when the variation is measured by molecular tools (e.g., microsatellites or single‐nucleotide polymorphisms). Some of this variation influences morphological, physiological, and other types of functional genetic variation that affect the performance of individuals and populations. Therefore, this “functional (non‐neutral) genetic variation” has important consequences for population dynamics, species interactions, and ecosystem function. Importantly, this functional variation might or might not be “adaptive,” that is, improving fitness (survival and reproduction). By contrast, “neutral (nonfunctional) genetic variation” has no such direct consequences and is more commonly considered as a proxy indicator for important population parameters, such as effective population size, gene flow, genetic integrity, or evolutionary potential (e.g., Parker, Snow, Schug, Booton, & Fuerst, 1998; Sunnucks, 2000).

Our goal was to explain the need for, and outline a strategy for, incorporating intraspecific variation into monitoring programs. We first illustrate how this variation promotes and maintains relevant levels of biodiversity, community integrity, and ecosystem function. We then outline how human‐induced environmental changes impact intraspecific variation, imposing environmental, economic, and social costs. Finally, we outline some potential monitoring strategies to observe and predict regional and global changes in intraspecific variation. Work along these lines would greatly increase our ability to predict, prevent, and mitigate detrimental ecosystem changes.

## 2 Intraspecific Variation Is Critical for Population Dynamics, Community Structure, and Ecosystem Function

Intraspecific variation is both the product of, and the foundation of, evolutionary and ecological processes (summarized in Table 1). Therefore, understanding the origins, architecture, and maintenance of genetic variation is critical for predicting the short‐ and long‐term responses of populations, communities, and ecosystems to novel and changing environments (Hendry et al., 2011; Lankau, Jørgensen, Harris, & Sih, 2011).

**Table 1:** Roles of intraspecific variation in ecological and evolutionary processes with representative open access articles

Levels	Processes	Summary	Examples of open access articles
Population	Portfolio effects	Genetic variation (and biodiversity) reduces risks and buffers negative impacts of changing environments. Individuals with various genotypes may produce a wide range of responses to the environment, thus contributing to population stability	Schindler et al. (2015) reviewed existing papers to illustrate the importance of diversity, both inter‐ and intraspecific variations for population persistence and evolutionary potentials
	Connectivity, effective population size, and mating success	Genetic variation increases effective population size and reduces risks of inbreeding depression, thus ensuring offspring survival	Hoffman et al. (2014) suggested that higher neutral genetic variation reduces the impact of inbreeding depression and the negative impact on population health
	Adaptability/evolvability	Genetic variation provides genotypes for new selections in a changing environment and contributes to populations fitting into the new environment	Merilä and Hendry (2014) reviewed evolutionary responses to climate changes. Additional examples of environmental changes are listed in the text
Community and ecosystems	Species diversityAbundancePrimary productivityPlant–soil interaction	Increasing genetic and phenotypic variations within species typically increases its primary productivity, species diversity, and abundance of mutualistic and antagonistic species (e.g., herbivores), and influences in plant–soil interactions	Crutsinger (2016) reviewed a number of examples illustrating how genetic variation influences the diversity and abundance of surrounding species, productivity, and plant–soil interactions
	Stability of ecosystem processes	Due to the above effects, genetic variation contributes to the stability of ecological processes and functions	Genung et al. (2010) found that the genetic variation of flowering species increases the floral abundance and number of visiting pollinators, thus ensuring the reproduction of the species and a sustainable food supply for pollinators

### 2.1 Effects on population dynamics

Environmental change will often harm populations that are poorly suited to the new conditions, which can lead to population declines, extirpation, and extinction (e.g., Green, Cornell, Scharlemann, & Balmford, 2005; Pörtner & Knust, 2007; Thomas et al., 2004). These negative effects can be offset, in part, when diversity within and among populations can help to buffer these problems through the so‐called portfolio effect (Leimu, Vergeer, Angeloni, & Ouborg, 2010; Moore, Yeakel, Peard, Lough, & Beere, 2014; Schindler, Armstrong, & Reed, 2015; Schindler et al., 2010). This effect predicts that higher biodiversity minimizes the overall risk in stability of ecosystem functions. In addition, populations can respond to detrimental environmental changes through migration to more optimal locations, adaptive plasticity, or evolutionary (genetic) adaptation. However, the first two options are often constrained to the point that evolutionary adaptation becomes a critical component of a species’ persistence in the face of environmental change (Phillimore, Had, Jones, & Smithers, 2012; Visser, 2008). Therefore, careful attention needs to be paid to how organisms evolve in response to environmental change.

Evidence for evolutionary adaptation to environmental change is widespread in a spatial context (i.e., populations in different environments show local adaptation to those environments), which reflects the action of past selection in shaping biodiversity (Schluter, 2000). However, these spatial patterns typically arise over long timescales, raising the question as to whether or not a similar adaptation can occur over much shorter timescales that typify human‐induced environmental change (Merilä & Hendry, 2014). The short answer appears to be “yes,” at least in some cases, in that a large number of studies have demonstrated adaptive evolution over time frames ranging from years to decades (reviewed in Reznick & Ghalambor, 2001; Hendry, Farrugia, & Kinnison, 2008). Such contemporary evolutionary responses have been observed in response to hunting/harvesting (Coltman et al., 2003; Pigeon, Festa‐Bianchet, Coltman, & Pelletier, 2016), pollution (Antonovics, Bradshaw, & Turner, 1971; Levinton et al., 2003), introduced species (Strauss, Lau, & Carroll, 2006), novel and changing climates (Bradshaw & Holzapfel, 2001; Colautti & Barrett, 2013; Merilä & Hendry, 2014), and novel environments (Prentis, Wilson, Dormontt, Richardson, & Lowe, 2008). At the same time, however, many other populations that have faced environmental change clearly did not evolve rapidly enough, as evidenced by frequent extirpations and extinctions (Barnosky et al., 2011; Hughes, Daily, & Ehrlich, 1997). Thus, the critical question becomes: What factors determine the potential for evolution to avert the extirpation of populations and the extinction of species threatened by human‐induced environmental change?

**Figure 1:**
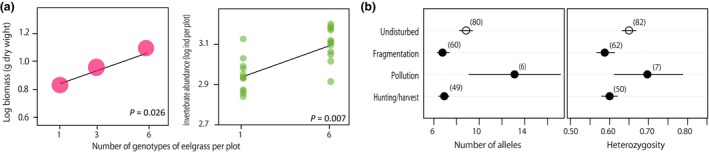
Roles of genetic diversity in ecosystems but dramatically influenced by human‐induced environmental changes. (a) Genetic diversity of eelgrass (*Zostera marina*) affected by ecosystem functioning and resilience (replotted from Reusch et al., 2005, Copyright (2005) National Academy of Sciences, USA). Experimental plots were designed with one, three, and six eelgrass genotypes and the mean biomass of eelgrass (left panel) and the number of invertebrates within each plot at the end of a 4‐month experiment were measured (right panel). (b) Shown are the results of a meta‐analysis by DiBattista et al. (2008) comparing variation in microsatellite markers between undisturbed populations and populations subject to various types of human disturbance (redraw from the original). Fragmentation and hunting/harvesting tend to decrease genetic diversity (left: number of alleles, right: heterozygosity), whereas pollution has less predictable effects. The number of studies is indicated in parenthesis

A critical determinant of the potential for adaptive evolution is the amount of genetic variation in fitness and, thus, in fitness‐related traits: a potential often assessed as additive genetic variance (*V*_A_) or heritability (*h* ^2^ = *V*_A_/*V*_P_), where *V*_P_ is phenotypic variance (Hoffmann & Merilä, 1999; Visscher, Hill, & Wray, 2008) as the concepts were initially introduced by Fisher (1930) and Wright (1920). In theory, the adaptive evolutionary rate is directly proportional to *V*_A_ (Fisher, 1930). However, given that *V*_A_ is not easily measured in natural populations (Kruuk, 2004), many studies have instead used molecular genetic diversity as a proxy for the overall functional and neutral genetic variations. Although molecular genetic diversity is not always correlated with functional variation in natural populations (Reed & Frankham, 2001), recent experimental studies have shown that molecular genetic diversity and genotypic diversity, the variation in genotypes among individuals, can predict population responses to environmental change (see Figure 1a; Vázquez‐Domínguez, Piñero, & Ceballos, 1999; Reusch, Ehlers, Hämmerli, & Worm, 2005; Hoffman et al., 2014). Moreover, recent innovations in molecular biology enable the direct assessment of functional genetic variants responsible for adaptation (discussed later in “What types of variation should be monitored?”). This ability augurs a new era for biodiversity monitoring with a more direct measurement of functional genetic variation, which is obviously most relevant to predicting evolutionary responses in many species facing the environmental changes.

### 2.2 Effects on communities and ecosystems

The theoretical and empirical studies have indicated that ecological system functionality and species interaction, which provides fundamental services for humanity, are affected by biodiversity (Hooper et al., 2005; May, 1973). Most studies addressing this topic focus on interspecific diversity (e.g., the number of species or functional groups); however, intraspecific variation can also be substantial and important in communities and ecosystems (Siefert et al., 2015). For example, increasing genetic and phenotypic variations within a species typically increases species diversity and abundance and primary productivity, promotes positive plant–soil interactions (reviewed in Crutsinger, 2016), and stabilizes ecosystem functions (Genung et al., 2010; Prieto et al., 2015). Although species and individual interactions due to variance in individuals are less predictable, these effects appear to be strongest when the species in question plays an important role in the ecosystem (Hendry, 2016; Hughes, Inouye, Johnson, Underwood, & Vellend, 2008); that is, it is a “keystone species,” “foundation species,” “niche constructor,” “strong interactor,” and so on.

Two primary mechanisms can explain the positive relationship between diversity (both among and within species) and various community/ecosystem processes (Loreau & Hector, 2001). First, diverse communities use a wider range of resources due to resource partitioning or positive species interactions (i.e., “complementarity effects”), which can increase productivity and nutrient cycling. For example, genetic variation in foundation tree species helps to maintain the diversity of associated plants, animals, and fungi because different tree genotypes are advantageous to different species interactions (Barbour et al., 2009; Zytynska, Fay, Penney, & Preziosi, 2011). Second, communities with greater diversity have a better chance of including a few key species that have large effects on ecosystem processes (i.e., “selection effects”). At the same time, it is important to recognize that greater diversity is not always “better,” such as when it compromises local adaptations (Hansen, Carter, & Pélabon, 2006) or allows one species to dominate and have detrimental effects on other species. The many ways that intraspecific variation influences community/ecosystem function and stability are context‐specific and are still being discovered (Hendry, 2016). Overall, then, diversity, both within and among species, is best thought of as providing not just “ecosystem services” but, more generally, “evosystem services” (Faith et al., 2010).

## 3 Human Activities Dramatically Influence Crucial Aspects of Intraspecific Variation

The many potential influences of intraspecific variation, as described above, motivate a need to consider how this variation is influenced by human activities, such as habitat loss and degradation, harvesting and hunting, pollution, species introductions, climate change, and so on. One major consequence is decline in populations and extirpations, which are estimated at three to eight times higher than baseline (Hughes et al., 1997). Another major consequence is that humans can directly (e.g., by hunting and harvesting) or indirectly (e.g., climate change and pollution) impose novel selective pressures that lead to evolutionary responses with potential negative effects on future stability, productivity, and persistence (Coltman et al., 2003; Law & Salick, 2005; Pespeni et al., 2013; Stockwell, Hendry, & Kinnison, 2003; Swain, Sinclair, & Mark Hanson, 2007). A third consequence is that human activities can increase the gene flow among populations (the opposite of reduced connectivity) and hybridization among species, which can cause biotic homogenization (McKinney & Lockwood, 1999), outbreeding depression (Dudash & Fenster, 2001), and speciation reversal (De León et al., 2011; Seehausen, Takimoto, Roy, & Jokela, 2008; Vonlanthen et al., 2012). These various effects indicate ways in which management decisions can have a direct influence on genetic variation and evolutionary potential (Santamaría & Mendez, 2012). Below, we illustrate some significant human‐induced environmental changes that affect genetic variation.

### 3.1 Habitat modification

Land use by humans (e.g., agriculture and settlements) is one of the most important drivers of global biodiversity loss (Ellis, Antill, & Kreft, 2012), partly through the above effects; decreased population size, novel selection, and increased gene flow. First, land use often leads to habitat loss for many species, which can reduce the size and connectivity of the affected populations. Typical outcomes include increased inbreeding and reduced intraspecific variation (Figure 1b; Aguilar, Quesada, Ashworth, Herrerias‐Diego, & Lobo, 2008; Allendorf, Luikart, & Aitken, 2012; DiBattista, 2008; Frankham, Ballou, & Briscoe, 2002), which can impact survival and reproduction by reducing portfolio effects (Bello‐Bedoy & Núñez‐Farfán, 2011; Hoffman et al., 2014; Keller & Waller, 2002; Núñez‐Farfán, Fornoni, & Valverde, 2007), increasing the mutation load (Agrawal & Whitlock, 2012), and hampering evolutionary responses to environmental change (Bijlsma & Loeschcke, 2012). Second, environments altered by land use often impose novel selective pressures, leading to occasionally large phenotypic and genetic responses that alter intraspecific variation. Examples include adaptation to industrial pollution by plants (Antonovics et al., 1971; Bratteler, Lexer, & Widmer, 2006), terrestrial insects (Clarke & Sheppard, 1966; Cook & Saccheri, 2012), and various aquatic organisms (Levinton et al., 2003). Third, habitat loss can lead to altered species interactions, which can increase hybridization. For example, habitat conversion has been followed by an increased chance of hybridization among gray wolves, *Canis lupus*, and coyotes, *C. latrans* (Koblmüller, Nord, Wayne, & Leonard, 2009; Lehman et al., 1991).

**Figure 2:**
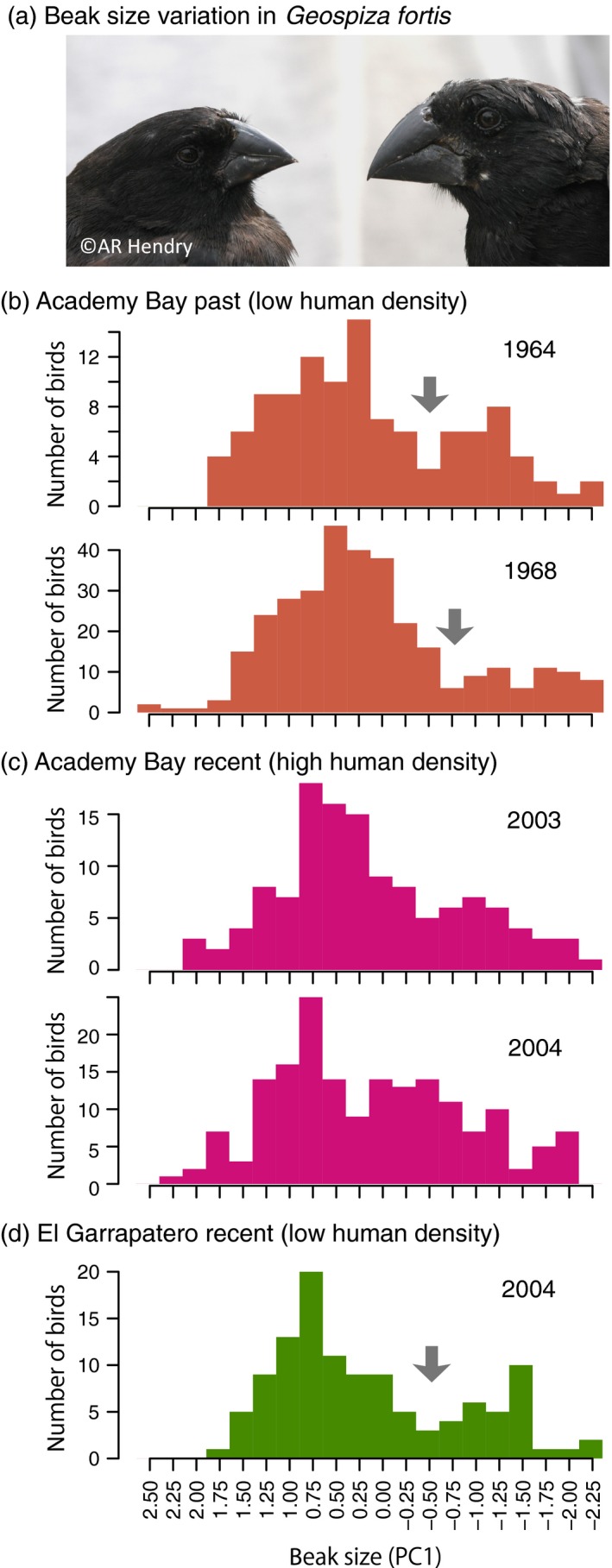
Human‐induced environmental changes affect selection. An example of human activities altering existing selection in (a) the ground finches, *Geospiza fortis*. (b) The degrees of bimodality in beak sizes within the medium ground finch were stronger in the absence of human influences in 1964 and 1968, (c) than in the presence of human influences in 2003 and 2004 at Academy Bay. (d) The strong bimodality persisted in 2004 at El Garrapatero when the human densities were still low. Gray arrows show discontinuities in beak size variation in the populations statistically confirmed to have the strong bimodality. Beak size variation was calculated as the first principal component (PC1) of the multiple size measurements. These data were replotted from Hendry et al. (2006)

The above examples follow naturally from the expectation that land use has a strong negative effect on species. However, land‐use changes can have seemingly positive or at least unanticipated effects on some species that nevertheless negatively impact diversity. For example, as illustrated in Figure 2, increased provisioning of human foods for Darwin's finches, *Geospiza fortis*, reduced the disruptive selection that had historically maintained distinct intraspecific beak size morphs (De León et al., 2011; Hendry et al., 2006). As another example, eutrophication caused by intensive agriculture (Tilman et al., 2001) could increase productivity but it also promotes hybridization, causing the collapse of “species flocks” into “hybrid swarms” (Seehausen, 1997; Vonlanthen et al., 2012). The consequences of land use are, therefore, complex and require careful monitoring of both natural selection (abiotic and biotic environmental changes) and the structure of intraspecific variation.

Further complexity comes from the many ways in which connectivity is invoked, considered, and managed. First, land‐use (and other anthropogenic) changes can increase connectivity in some cases and decrease it in others, often dramatically either way. Second, the resulting increasing or decreasing gene flow (and hybridization) can have both positive and negative influences on populations and species (reviewed in Garant, Forde, & Hendry, 2007). Gene flow can increase an effective population size, reduce inbreeding, aid adaptive responses to environmental change, and spread adaptive variants among populations, while it reduces genetic differences among populations which can limit portfolio effects and hamper local adaptation by introducing maladapted genes from other environments (García‐Ramos & Kirkpatrick, 1997; Lenormand, 2002; Slatkin, 1987). Given that changes in connectivity and a release of translocated or captive raised individuals are one of the more easily implemented management actions (e.g., “genetic restoration” and “assisted migration”), these various effects are often debated both theoretically and practically (Aitken & Whitlock, 2013; Laikre, Schwartz, Waples, & Ryman, 2010; Santamaría & Mendez, 2012).

### 3.2 Climate change

Human‐induced climate change is altering patterns of temperature, precipitation, erosion, and ocean acidification (IPCC 2013). As with other human influences, these consequences of climate change can result in decreased population size, novel selection, and increased gene flow, which can then alter interspecific variation. Changes in population size are a common outcome of climate change, including declines in many species (Both, Bouwhuis, Lessells, & Visser, 2006), but also in increases for other species (Massimino, Johnston, & Pearce‐Higgins, 2015; Rochlin, Ninivaggi, Hutchinson, & Farajollahi, 2013). These changes in abundance can then have various effects on intraspecific variation, as already discussed above. Changes in selection are also common, most obviously in relation to the timing of key life history events (Merilä & Hendry, 2014). For instance, evolutionary responses to climate‐induced selection have been documented for the flowering time in mustards, *Boechera stricta* (Anderson, Inouye, Mckinney, Colautti, & Mitchell‐olds, 2012), life history timing in pitcher plant mosquitoes, *Wyeomyia smithii* (Bradshaw & Holzapfel, 2001), and reproductive timing in red squirrels, *Tamiasciurus hudsonicus* (Réale, McAdam, Boutin, & Berteaux, 2003). Finally, connectivity is often influenced by climate change through the nearly ubiquitous and sometimes dramatic changes in species’ distributions (Bálint et al., 2011; Parmesan, 2006; Pauls, Nowak, Bálint, & Pfenninger, 2013), including range contractions (Habel, Rödder, Schmitt, & Nève, 2011) and range expansions (Hewitt, 1996). These shifting distributions lead to many alterations in species interactions that can also instigate hybridization (Garroway et al., 2010; Mimura, Mishima, Lascoux, & Yahara, 2014). Thus, as for habitat loss, altered (and actively altering) connectivity is frequently discussed with regard to potential management actions in response to climate change (Aitken & Whitlock, 2013; McLachlan, Hellmann, & Schwartz, 2007).

### 3.3 Harvesting and domestication

Harvesting and domestication can reduce the size of wild populations and thereby alter genetic variation in many of the ways described above (e.g., Allendorf, England, Luikart, Ritchie, & Ryman, 2008; Harris, Wall, Allendorf, Harris, & Wall, 2002). However, many harvested and domesticated species are abundant enough that the problems associated with a small population size are often negligible. Instead, the most obvious effect of harvesting and domestication is typically altered selection. For instance, hunting and fishing practices can inadvertently result in selection for a smaller body size and earlier maturation (Coltman et al., 2003; Law & Salick, 2005; Swain et al., 2007), which can negatively influence survival, resilience, and recovery (Fenberg & Roy, 2008). Like harvesting, domestication (e.g., crop maturity and fish hatcheries) can alter selection and lead to evolutionary changes that alter productivity (Denison, 2012). Domestication can also lead to sustained genetic bottlenecks that dramatically decrease genetic variation (Doebley, Gaut, & Smith, 2006), which can then negatively impact the remaining wild populations through competition or gene flow. For instance, genetic change in captive‐reared fish populations that are then released in the wild can reduce the reproductive potential of natural populations (Araki, Berejikian, Ford, & Blouin, 2008). Importantly, the above effects can persist long after the human activity ceases. For example, harvested silverside fish (*Menidia menidia*) populations can have evolutionary reductions in growth rate and body size that persist for decades after harvesting is halted (Conover, Munch, & Arnott, 2009).

### 3.4 Species introductions

Species introductions into new geographic areas sometimes lead to species “invasions” that can reduce the abundance of native species through competition, predation, hybridization, and infection (Pyšek & Richardson, 2010). Again, the above‐described effects of declines in populations can happen to native species. In addition, phenotypic and genetic changes have been observed in many introduced species and the native species with which they interact (Hendry et al., 2008; Mooney & Cleland, 2001). Although the extent to which these changes are adaptive is not always certain (Colautti & Lau, 2015), evolution in introduced species is predicted to influence the rate, extent, and impact of invasions (García‐Ramos & Rodríguez, 2002; Vázquez‐Domínguez, Suárez‐Atilano, Booth, González‐Baca, & Cuarón, 2012). Thus, novel selective pressures can lead to evolutionary changes in both native and invasive species that then influence the abundance of those species, with expected further consequences for intraspecific variation. Although these effects are typically assumed to be negative for native species, the opposite can sometimes also occur, such as when a native species benefits from the introduction of new food resources (Carroll et al., 2005). The result can be a decrease in intraspecific variation owing to increased gene flow (as in the aforementioned Darwin's finch example, Figure 2), or an increase in intraspecific variation owing to the formation of new insect host races (Drès & Mallet, 2002).

## 4 Toward a Monitoring System for Intraspecific Variation

**Figure 3:**
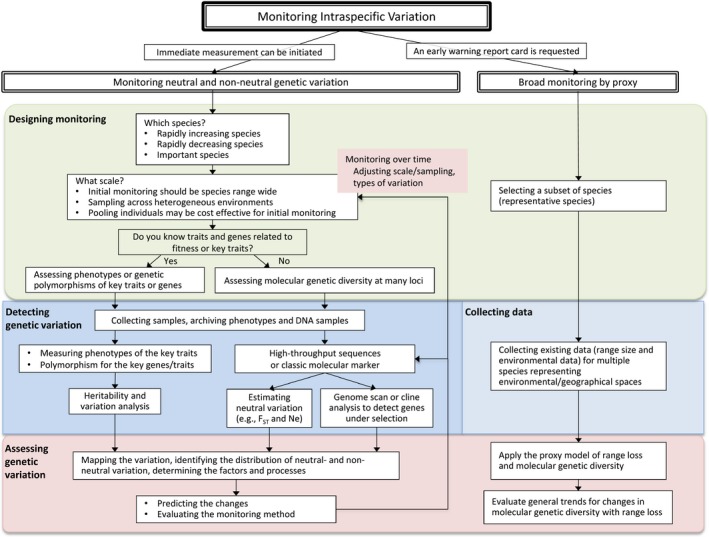
A suggested flowchart for monitoring intraspecific variation

We have highlighted two basic points: (i) Intraspecific variation has important consequences for population dynamics, community structure, and ecosystem function; and (ii) intraspecific variation is strongly influenced by human‐induced environmental change. From the intersection of these two points comes the need for a monitoring program that can track and assess ongoing changes in intraspecific variation and thus provide important baseline data for assessing the consequences for populations, communities, ecosystems, and human well‐being. Our goal in the rest of this study was to highlight some elements that an appropriate monitoring program might include. We suggest two main strategies: (i) explicit empirical monitoring of variation for selected species and (ii) modeling variation for a larger set of species (illustrated in Figure 3). The first strategy requires measurements of intraspecific variation, ideally including *functional variation*, in combination with environmental parameters. As the intensive effort required might not be feasible for many species, the second strategy, which is less data‐intensive, uses models to predict how variation will be lost based on environmental changes.

### 4.1 Monitoring variation for specific target species

#### 4.1.1 Which species should be monitored?

As it is obviously impossible to monitor all species, we need to select specific species for monitoring, presumably either those of a direct conservation or management interest or those that can act as “indicator species.” For instance, suitable indicator species have been suggested to be those that respond rapidly to environmental change over short timescales and that have strong effects on ecosystem function (Pereira & David Cooper, 2006). Along these lines, the Group on Earth Observations Biodiversity Observation Network (GEO BON 2010) defined three main categories of species that might be appropriate to monitor, which we summarize as (i) *rapidly declining species*, (ii) *rapidly increasing species*, and (iii) *important species*. Here, we focus on these categories and describe some successful examples of their monitoring during human‐induced environmental change. In cases where causal inferences are desired, it could also be valuable to track genetic changes in “control” species that are not rapidly increasing, rapidly decreasing, or “important.”

*Rapidly declining species* are typically listed as critically endangered on the IUCN Red List or by the Evolutionarily Distinct Globally Endangered Program (EDGE, http://www.edgeofexistence.org). The loss of variation in such species could reflect—and contribute to—a decline leading to extinction. Such species are typically of intense interest and are therefore frequently monitored for various aspects of intraspecific variation. As one example, a comparative genomic study across avian species discovered that both the loss of genetic variation and the accumulation of deleterious mutations of protein‐coding genes contributed to major genetic defects in endangered species (Li et al., 2014). They also implied that some sets of genes could contribute to averting extinction or enhancing recovery in endangered crested ibis (*Nipponia nippon*) populations. All endangered and rapidly declining species would benefit from similar assessments and monitoring.

Typical examples of *rapidly increasing species* include many invasive alien species, novel pests, and emerging infectious diseases (EIDs). These species are important to monitor, not only because of their negative impacts on biodiversity and human well‐being, but also because their rapid increases often have clear genomic signatures. For example, introduced yellow monkey flower (*Mimulus guttatus*) populations show a reduced neutral genetic variation but also signatures of positive selection at genomic regions linked to flowering time and stress responses (Puzey & Vallejo‐Marín, 2014). Similarly, genetic elements that enhance invasiveness have been tracked by comparing molecular genetic diversity between introduced and native populations of an ant species, *Cardiocondyla obscurior* (Schrader et al., 2014), and a wetland grass species, *Phalaris arundinacea* (Lavergne & Molofsky, 2007). Importantly, the genomic signatures that point toward invasions or EIDs can sometimes be detected before the expansion begins in earnest, and so, monitoring of introduced species can provide an early‐warning system for upcoming challenges. Given that rapidly increasing species can be widespread, international collaborations will often be required for such monitoring. A good example of this is the “Global Garlic Mustard Field Survey” (http://www.GarlicMustard.org/) that collects field data and seeds of this invasive species.

*Important species* could be commercially important (fisheries, forestry, or agriculture), ecologically important (keystone, foundation, or ecosystem engineer), or culturally important (including “flagship” species that attract considerable public attention). These species are also considered in CBD Aichi Biodiversity Targets (i.e., Target 13). Monitoring such species is critical not only due to their importance but also because that importance can have consequences for intraspecific variation. For instance, species under cultivation can suffer from bottlenecks, strong selection, and the propagation of specific strains that reduce intraspecific variation and thereby have negative consequences for safeguarding food security and for “option values” (Jump, Marchant, & Peñuelas, 2009). An existing application of this thinking is the ongoing effort by agronomists to collect and evaluate landraces and wild relatives of crop species (Hyten et al., 2006; Plucknett, Smith, Williams, & Anishetty, 1987), and to find a source of new genetic markers for future improvements. Another example is the monitoring of changes in life history traits in the commercially harvested fish species, Atlantic cod, *Gadus morhua* (Olsen et al., 2005), which can signal disastrous collapses of fisheries and thereby perhaps motivate mitigating management actions.

#### 4.1.2 What types of variation should be monitored?

##### Neutral genetic variation

Human‐induced factors that are likely to affect genetic variation include decreased population size, novel selection, and increased gene flow (as previously described). Neutral molecular markers can be used to detect changes in population size and gene flow (Table 2) through the estimation of key parameters of genetic variation, such as the number of alleles (A), heterozygosity (He), effective population size (*N*_e_), inbreeding (*F*), and population divergence (*F*_ST_). As theoretical and empirical studies suggest (Leberg, 2002; Nei, Maruyama, & Chakraborty, 1975; Spencer, Neigel, & Leberg, 2000), the number of alleles (A) can be a more sensitive indicator of population decline than heterozygosity (He) in a variety of scenarios (Hoban et al., 2014). Effective population size (*N*_e_) is also an important indicator for monitoring changes in population size, and several statistical methods to estimate *N*_e_ have been developed and evaluated for their efficacy in early detection of population declines (e.g., Antao, Pérez‐Figueroa, & Luikart, 2011). Although translating the population divergence parameter (*F*_ST_) as an indirect measurement of gene flow is unrealistic (Whitlock & Mccauley, 1999), several statistical approaches using various molecular markers allow a relatively accurate estimation of migration rate, many of which use coalescence‐based Bayesian approaches (e.g., Beaumont, 2010; Beerli & Palczewski, 2010; Hey, 2010; Wilson & Rannala, 2003). These measures are considered to be relatively affordable and rapid surrogate measures of key population genetic parameters. Such markers certainly can be a key component of any monitoring program, and appropriate methods have been frequently reviewed elsewhere (e.g., Luikart, Sherwin, Steele, & Allendorf, 1998; Schwartz, Luikart, & Waples, 2007). Given this existing literature, here we focus more directly on monitoring functional (non‐neutral) variation, which may have significant impacts on population persistence and therefore management options for species exposed to novel selective pressures.

**Table 2:** Examples of techniques currently most adequate for monitoring genetic variation and the impacts of human‐induced environmental changes. The cost for these techniques is constantly changing; thus, it is not included

Examples of suitable techniques for monitoring	Types of variation	Advantages	Disadvantages	Detection of human‐induced changes	
				Population size/gene flow (neutral variation)	Selection (non‐neutral variation)
Genetic monitoring					
Traditional molecular markers					
Codominant markers (e.g., microsatellites)	Mostly neutral	Cheap and reproducible. Microsatellites are usually hypervariable. Codominant markers. Multiplex PCR (up to 10‐12 loci) is cost‐effective	Often requires species‐ or genera‐specific primer information. Location of the markers on the genome is usually unknown. Often limited number of loci (10‐100 loci). Only polymorphic loci is used, which may cause biased estimations. Poor‐levels of interlaboratory calibration	Yes, but using only polymorphic loci can lead to biased estimation	Limited, depending on the number of loci
Dominant markers (e.g., AFLP—amplified fragment length polymorphism)	Mostly neutral	Genome information not required. Relatively cheap. Highly polymorphic at hundreds of loci	Less reproducible. Location of the markers on the genome is usually unknown. Dominant markers. Only polymorphic loci is used, which may cause biased estimations. Poor‐levels of interlaboratory calibration	Yes, but using only polymorphic loci can lead to biased estimation	Limited, depending on the number of loci
SNPs and sequences					
Sanger DNA sequencing	Neutral and functional	Useful for DNA barcoding. Suitable if genes responsible for key traits are known prior to monitoring. Low error rate and high data transferability between laboratories	Lower polymorphism than traditional molecular markers. Mostly species‐ or genera‐specific for functional genes; thus, genome or primer information is required	Limited, due to low polymorphism if few loci are available	Yes, with prior information of the target loci associated with key traits
SNP chips	Neutral and functional; mostly functional for qPCR‐based SNP chips	Array of genes allows screening of candidate loci. Can deal with hundreds to 10^5^ loci. Low error rate and high data transferability between laboratories	Require prior genome information for constructing arrays. Only polymorphic loci is used, which may cause biased estimations	Limited, due to potentially nonneutral polymorphisms	Yes, effective screening of the candidate loci from a set of genes
Reduced representative sequencing (RRS) (e.g., genotyping‐by‐sequencing and RADseq)	Neutral and functional	10^4^ to 10^5^ loci detected from genome‐wide in nonmodel organisms. Multiple individuals can be processed at once. A reference sequence is not required, but preferable for annotating DNA and some analyses. With enough read depth, low error rate and high data transferability between laboratories	Representing roughly 1% of genome sequences. Putative functions of loci are often unknown if reference sequences are not available	Yes, with increased accuracy	Yes, allows the genome‐wide screening to identify candidate loci from anonymous or annotated DNA
Whole‐genome resequencing	Neutral and functional	(Almost) complete genome polymorphism. Unbiased estimation of population genetic parameters. With enough read depth, low error rate and high data transferability between laboratories	Data often overkill for standard population estimations (e.g., *F*_ST_ and *N*_e_). Relatively expensive. Requires genome information (e.g., reference sequences)	Yes, data is often more than needed but best accuracy is obtained without subsetting a set of loci	Most appropriate. Allows scanning the whole genome to identify the candidate loci
Phenotype monitoring					
Trait measurement					
Trait variance, heritability and additive genetic (co)variance of traits	Phenotypic, neutral, and functional	Provide direct evidence for how the traits might respond to environmental changes. Provide crucial information for interpretation of genomic data	Labor‐ and cost‐intensive to measure phenotypes in wild populations. Subject to genotype–environmental interaction. Often require common garden experiments to identify genetic‐based variation	No, not accurate estimation	Yes, direct evidence in association with survival, growth and reproduction (fitness)
Gene expression					
Quantitative PCR (qPCR), RNAseq, qPCR‐based DNA chips	Mostly functional (expressing genes)	Useful to detect changes in gene expression of target genes or a whole genome	Require prior information for genes already known to have important influences on (fitness‐related) traits	No	Allow measurements of changes in expression levels of fitness‐related genes among environments

##### Functional variation

Monitoring variation in ecologically important and heritable phenotypes (e.g., phenology, growth, and physiology) provides direct evidence for how those traits might respond to environmental change. For instance, many long‐term studies have tracked changes in mean phenotypes to infer the effects of disturbances such as in fisheries (Kuparinen & Merilä, 2007), hunting (Coltman et al., 2003; Pigeon et al., 2016), or with climate change (Parmesan, 2006). Some studies have also shown changes in trait variance, such as declining variance in body size over nine decades in fished cod populations (Olsen, Carlson, Gjøsaeter, & Stenseth, 2009). Of additional interest are the quantitative genetic parameters underlying these changes, such as heritability and additive genetic (co)variances estimated from controlled breeding experiments or “animal model” analyses of intensively monitored and pedigree populations (Bock et al., 2014; Charmantier & Garant, 2005; Charmantier, Perrins, McCleery, & Sheldon, 2006; DiBattista, Feldheim, Garant, Gruber, & Hendry, 2011; Merilä & Hendry, 2014; Wilson et al., 2010). These approaches can often reveal temporal changes in quantitative genetic parameters and the contribution of genetic and plastic effects to temporal trait changes. At the same time, they can be extremely labor‐intensive and subject to strong complications from genotype‐by‐environmental interactions. For example, Kellermann, van Heerwaarden, Sgrò, and Hoffmann (2009) showed that tropical rainforest *Drosophila* species had a very low ability to evolve desiccation resistance in response to a reduced humidity (10% relative humidity) expected under environmental change, whereas van Heerwaarden and Sgrò (2014) showed that the same species had a high evolvability of desiccation resistance when tested under a more realistic reduction in humidity (35% relative humidity).

An alternative to working with phenotypes is to quantify variation in functional molecular loci (or other physically linked markers) expected to be under strong selection in the case of environmental change. For instance, in some cases, particular alleles in particular genes are known to be differentially sensitive to different temperatures (e.g., Feder & Hofmann, 1999) or salinities (e.g., Munns & Tester, 2008), and so on. In particular, key phenotypes can be controlled by complex nonadditive genetic mechanisms. Thus, understanding the effects of those genes on phenotypes is often essential for understanding the effects of genetic variation on population performance and its consequential effect on ecosystems. Therefore, quantifying the variation in these genes can reveal the evolutionary potential. In addition to monitoring sequence variation in functional genes, one can also monitor gene expression, such as through real‐time PCR (Satake et al., 2013). Of course, these methods are limited to genes already known to have important influences, and the amount of fitness variance explained by such genes is often very low.

An alternative to focusing on specific genes expected *a priori* to be under strong selection is that one can employ next‐generation sequencing (NGS) to genotype tens to hundreds of thousands of loci in hundreds of individuals at reasonable cost. The usefulness of genomic approaches has received increased attention for its value in conservation biology (reviewed in Allendorf, Hohenlohe, & Luikart, 2010; Shafer et al., 2015). Some common NGS approaches include whole genome resequencing, reduced‐representation sequencing (RRS), and pooled DNA sequencing (Pool‐seq). RRS methods include genotyping‐by‐sequencing (GBS; Davey et al., 2011), restriction site‐associated DNA sequencing (RADseq; Baird, Etter, Atwood, & Currey, 2008; Peterson, Weber, Kay, Fisher, & Hoekstra, 2012), and multiplexed ISSR genotyping‐by‐sequencing (MIGseq) (Suyama & Matsuki, 2015). By restricting sequencing to a fraction of the genome (e.g., restriction enzyme sites ~1% of the genome), GBS and RADseq can typically generate tens of thousands of polymorphisms for monitoring variation. These approaches do not require a pre‐existing reference genome, although availability of such a genome allows for more accurate calls for SNPs and a better ability to infer selection (discussed below). Pool‐seq (Futschik & Schlotterer, 2010) combines DNA from multiple individuals into a single sequencing run, which greatly reduces the cost of obtaining allele frequency data but sacrifices information about the linkage among genetic polymorphisms. Finally, RNA‐seq can be used to measure expression differences across thousands of genes without relying on *a priori* assumptions about which genes are important.

With enough loci generated by GBS, one can statistically partition loci into genomic regions or—when lacking a reference genome—”markers” that are neutral and regions that are under selection (Vitti, Grossman, & Sabeti, 2013). The former loci can be used to infer population genetic parameters such as those described at the beginning of this section, whereas the latter loci can be used to monitor functional markers. When a reference genome is available, one set of statistical approaches uses the linkage disequilibrium to infer selective sweeps on the genome (e.g., Sabeti et al., 2002). Another set of statistical approaches uses the distribution across loci as measures of population differentiation (e.g., *F*_ST_) to infer candidate loci under selection in a Bayesian framework (Beaumont & Balding, 2004; Foll & Gaggiotti, 2008). Moreover, a set of approaches looks for spatial or temporal associations among alleles at particular loci and environmental variables (e.g., temperature, pollution, fishing, hunting, and land use) thought to impose selection on organisms (Joost et al., 2007; de Villemereuil & Gaggiotti, 2015). As each of these approaches has its own strengths and weaknesses, it is commonly suggested that multiple methods be used for optimal inference (Hansen, Olivieri, Waller, & Nielsen, 2012).

In summary, it is clear that no single parameter is a sufficient metric of intraspecific variation. Instead, different parameters yield different insights and their combination is necessary for robust inferences (Table 2). For instance, by combining genotypic and phenotypic data of Atlantic salmon (*Salmo salar*), Barson et al. (2015) found a sex‐dependent dominance of the gene affecting age maturity; a heterozygote induces late maturity in females and early maturity in males. This finding in the genetic mechanism controlling the key traits may have significant impact on the population managements where the harvesting consequentially selected for early maturation (e.g., Olsen et al., 2005). Initial monitoring of genetic variation in broader contexts with multiple parameters is crucial for understanding the underlying mechanisms that determine the population performance. Hence, monitoring of both neutral and functional genetic variations and phenotypic changes may be necessary to effectively detect and interpret the impact of environmental/management changes on genetic variation. However, linking functional variation in changing environments to population viability and persistence is a challenging task. To aid such integration, we encourage field sampling protocols that archive phenotypes and DNA in ways that allow both current and future analyses depending on changes in resources and technologies. Obvious examples of such protocols are photographs, archiving well‐documented, complete, or partial specimens (such as fish scales or wood samples) in museums and herbaria (assuming that collecting specimens for preservation do not compromise the viability of the population), and long‐term preservation of DNA and RNA.

#### 4.1.3 What spatiotemporal scales should be monitored?

The simplest approach to predicting responses to environmental change would be a single‐time spatial survey relating intraspecific variation to environmental variation. This “space‐for‐time” substitution approach can be informative but many factors, most obviously different timescales, can dictate that spatial patterns will not always accurately predict temporal responses to environmental change (Fukami & Wardle, 2005; Merilä & Hendry, 2014). In short, temporal monitoring of populations is an essential component of any attempt to understand and predict how intraspecific variation will change with ongoing environmental change (Hoban et al., 2014; Schwartz et al., 2007).

#### 4.1.4 What timescale of monitoring is necessary?

The answer here will be species‐specific, taking into account information on life histories and generation times, and also environment‐specific, taking into account information on the timescale and “color” (autocorrelation) of environmental variation (e.g., de Barba et al., 2010; Dowling et al., 2014; Gotanda & Hendry, 2014; Hansen et al., 2012; Schwartz et al., 2007). At the very least, a few generations are necessary to reliably infer trends; however, many studies have found that considerably longer time frames are needed for reliable inference. For example, evolutionary responses of bighorn trophy rams to harvesting (Coltman et al., 2003), and red squirrels to climate change (Réale et al., 2003) that were initially inferred from at least a decade of data, were found to be quite different in the following decade (Pigeon et al., 2016). Simulation studies suggested that even in a 90% decline in population size it might be difficult to detect a signature in sensitive parameters (i.e., number of alleles) within a few generations (Hoban et al., 2014). Long‐term monitoring is therefore optimal but is also not feasible for many organisms. Fortunately, retrospective sampling (e.g., herbaria, museums, sediments, fish scales, or otoliths, and seed banks) can sometimes greatly extend the monitoring timescale by providing insights into past genotypes and phenotypes (Morinaga, Iwasaki, & Suyama, 2014; Wandeler, Hoeck, & Keller, 2007). In some cases, seeds and eggs are still viable over decades, allowing for the “resurrection” of past genotypes for direct comparison of current genotypes (Angeler, 2007; Franks et al., 2008) Finally, in long‐lived organisms, comparative evaluations of genetic variation patterns in adults and juveniles can provide an “early warning” of potential genetic changes (Kettle, Hollingsworth, Jaffré, Moran, & Ennos, 2007; Lowe, Cavers, Boshier, Breed, & Hollingsworth, 2015).

#### 4.1.5 What spatial scale of monitoring is necessary?

If all populations across a species range experienced the same environmental change and responded similarly to that environmental change, then monitoring a single population would be sufficient. However, environmental changes vary dramatically across species’ ranges and different populations respond differently even to the same environmental change (Both & Visser, 2001; Hampe & Petit, 2005). Thus, the optimal monitoring strategy would take into account the spatial grain of environmental change and the spatial grain of population responses to a given environmental change.

For most species, a range‐wide assessment first needs to be conducted so as to understand broad‐scale intraspecific variation and how it is structured within and among populations in relation to spatial (distance) and environmental variations. These assessments serve multiple purposes as they point to key factors, such as connectivity and gene flow (and the potential for portfolio effects), associations between environments and genotypes/phenotypes (the value of which was noted above), and which specific populations contain particularly high or low amounts of within‐population diversity or provide particularly important contributions to among‐population diversity. For instance, populations in the core part of a species’ range often, but not always, contain the most within‐population genetic variation, whereas those from the range peripheries are often, but not always, the most distinct from other populations and also the most susceptible to population size changes (Hampe & Petit, 2005; Smith & Steyn, 2005).

Information from range‐wide assessments can then be used to select particular populations for temporal monitoring. This selection can be aided by systematic methodologies to adjust (scale‐down) geographic observation scales for population sampling to those that best describe spatial trends in genetic variation within a species (Pauls et al., 2013; Pfenninger, Bálint, & Pauls, 2012). Decisions of which populations to monitor can also depend on specific policy and management concerns, locations where environmental change is greatest, particularly unique populations, populations expected to play a major role in range expansions or contractions, and many other considerations. For an invasive species, sampling that covers both the native and the introduced range can trace the invasion and capture evolutionary changes from the ancestral state.

#### 4.1.6 How intensive should sampling be within populations?

For severely endangered species that have only a few individuals remaining, an optimal strategy could be “complete genotyping (or ubiquitous genotyping),” that is, genotyping all individuals. An ongoing and successful example of this approach is the program in Japan where all individuals of more than 20 critically endangered plant species have been recorded and genotyped with microsatellite markers (Isagi & Kaneko, 2014). For larger populations, the minimum strategy should be to obtain good estimates of allele frequencies, for which sample sizes of 30–50 are typically sufficient (Dale & Fortin, 2014; Nei, 1978) and individual‐level data are unnecessary, such as in Pool‐seq (Futschik & Schlotterer, 2010). However, many questions benefit greatly from phenotyping and genotyping large numbers of individuals on fine spatial scales. For instance, individual‐level data allow the estimation of linkage disequilibrium, which facilitates estimates of effective population size as a sensitive indicator of population declines (Antao et al., 2011). In addition, Anderson et al. (2010) suggested that sampling resolution should be smaller than dispersal distances and home ranges so as to best evaluate population connectivity. Finally, high‐resolution individual‐level sampling better covers heterogeneous environments within the selected geographic area (Anderson et al., 2010; Oyler‐McCance, Fedy, & Landguth, 2012; Prunier et al., 2013).

#### 4.1.7 Assessing, evaluating, and improving the monitoring

The scope of empirical sampling will inevitably be incomplete for all but a few species. Fortunately, spatial simulation approaches in landscape genetics can help to cope with heterogeneity and incompleteness and can identify factors affecting genetic variation (Epperson et al., 2010; Landguth, Cushman, & Balkenhol, 2015). For instance, simulations have successfully identified agents that restricted gene flow in heterogeneous environments for American pikas, *Ochotona princeps* (Castillo, Epps, Davis, & Cushman, 2014), and American marten, *Martes americana* (Wasserman, Cushman, Schwartz, & Wallin, 2010). Simulations also identified the spread of adaptive genetic variation with range expansion (White, Perkins, Heckel, & Searle, 2013) and projected genetic consequences of future climate scenarios (Wasserman, Cushman, Littell, Shirk, & Landguth, 2013). It should be noted that evaluating monitoring methods (e.g., number of samples and sampling locations) is urgently needed to develop better monitoring methods, especially with new molecular techniques and complex ecological contexts. Simulation modeling with empirical data collected by initial monitoring helps to evaluate monitoring methods (Balkenhol & Fortin, 2015). Such efforts are greatly aided by the recent development of software to simulate genetic consequences in complex ecological contexts (reviewed in Hoban, 2014; Landguth et al., 2015). For instance, spatially explicit simulation software such as SPLATCHE2 (Ray, Currat, Foll, & Excoffier, 2010) and CDPOP (Landguth & Cushman, 2010) can use genetic data on heterogeneous landscapes to evaluate landscape resistance and range expansion in the context of environmental change. The utility of such approaches will escalate with the increasing availability of environmental data, including climate variables (e.g., Worldclim; http://www.worldclim.org) (Hijmans, Cameron, Parra, Jones, & Jarvis, 2005) and digital elevation model (DEM) variables, with free programs for geographic information systems (e.g., Quantum GIS, http://qgis.org).

### 4.2 Broad monitoring of representative species by proxy

Direct empirical monitoring, including simulations based on incomplete empirical data, will not be feasible in some cases, such as species that are very rare or hard to catch or genetically uncharacterized species. Moreover, detailed monitoring may not be the first choice if it detracts from the immediate needs for action to prevent extinction (Lindenmayer, Piggott, & Wintle, 2013). In such cases, simple predictive models that are broadly applicable across many species can provide some rapid broad‐brush insight. Indeed, global monitoring programs such as GEO BON wish to report regularly on such a global report card. One classic model‐based approach relates changes in population size (or habitat area as a proxy) to changes in genetic variation (Allendorf, 1986; Boecklen, 1986; Boyce, 1992). Such a model seems reasonable based on the established observation that species with larger population sizes or ranges have greater variation (Ellstrand & Elam, 1993; Frankham, 2012). We will now illustrate one way in which such a model might be implemented, but the specific model is intended to be only that—an illustration—and more sophisticated models should be developed, with some ideas introduced below.

One good initial candidate for general “proxy” model is a power law relationship, (*G*_0_/*G*_1_) = (*R*_0_/*R*_1_)^z^, where *G* is the genetic variation and *R* is the range extent; the subscript 0 indicates the original value and the subscript 1 indicates the new value (Neto, de Oliveira, Rosas, & Campos, 2011; also see Rauch & Bar‐Yam, 2004). Box 1 presents this approach schematically by showing how the power law can be used to predict the loss of genetic variation as a function of the loss of range area, while making a number of simplifying assumptions (e.g., random loss of area from a species’ range). Likely violations of these assumptions indicate that real species in real landscapes can deviate considerably from the simple prediction by having either a greater or lesser than expected loss of genetic variation for a given loss of range area. Thus, accurate predictions for specific species would require additional information and more sophisticated models that avoid some of the simplifying assumptions. However, the simple power curve might adequately capture the *average* outcome when calculated across many species and so provide a useful early‐warning report card of the general expected losses of within‐species genetic variation.

This strategy, based on range loss, considers consequences for neutral variation, but might not be effective at estimating changes in non‐neutral variation, such as that reflecting adaptation to environmental variation across the range of a species. More relevant predictions could be generated by employing information (for many species) on actual species distributions and estimated habitat/environmental loss (from Geographical Information System records or the “Map of Life”) to estimate how much of a species’ “environmental space” is lost. The “environmental diversity” or “ED” method (Faith, Ferrier, & Walker, 2004; Faith & Walker, 1996) can be applied to estimate fractional genetic variation loss for a given pattern of loss of sites in a species’ environmental space. Depending on whether losses in species’ environmental space are spread out or “clumped,” the same fractional loss of sites in the species’ environmental space can mean a high or low loss of functional genetic variation (Box 1). Similar approaches could be used for neutral genetic variation, providing an alternative to the simplifying assumption of random loss of range.

Box 1 **Proxies for within‐species genetic variation**

1 There is some evidence in support of a power law model linking within‐species genetic diversity loss to range loss (e.g., Neto et al., 2011). A power law model linking range and genetic variation is also supported, at least indirectly, by Morlon et al. (2011), who found support for a power relationship between the amount of phylogenetic diversity (PD) sampled and the sampled area. As an example, we have explored such a model through an analysis of genetic variation and range data from Alsos et al. (2012). They examined genetic data for 27 plant species over many populations covering the range of each species. They linked scenarios of range‐area loss to genetic variation loss through a random sampling approach. For a given species, they randomly removed an increasing number of grid cells (from the distribution of the species) and recorded the loss of alleles (loss of genetic variation). They repeated this random sampling 1000 times to find the median number of alleles (i.e., markers) lost for any given total number of grid cells removed (total range‐area lost). Based on these data, we explored the power curve relationship model relating the range extent loss to the genetic variation loss for each of the 27 species. While a power curve model consistently provided good fit, the power curve z values (see formula in main text) varied among these models, suggesting that there were difficulties applying one general model to predict genetic variation loss, given only fractional range‐loss information, over any set of representative species. However, the z values varied somewhat predictably (Figure a) according to the estimated population differentiation value (*F*_ST_) of the species (*F*_ST_ values provided in Alsos et al., 2012).

This suggests that a report card on the loss of genetic variation based on loss of geographic range extent for a given representative set of species is possible, if we have some estimates of genetic differentiation (e.g., estimates of *F*_ST_). A practical strategy may be the use of two pooled models for species broadly categorized as having large versus small differentiation values. Analysis of the Alsos et al. (2012) data suggests that this may be an effective, simple option (Figure b). Such a broad categorization also allows for expert opinion and other sources of information (such as dispersal or life history information) to be used to categorize species. Future work could explore whether a power curve with an intermediate z value (e.g., of 0.25) provides a robust proxy approach applicable to the tens of thousands of species in the Map of Life.

An alternative approach is possible when we have information, for a given species, about the pattern of losses of its distribution of sites in an environmental space (Figure c).

**Figure** Analysis of the genetic variation and range data from Alsos et al. (2013). (a) Z values from significant power curve models (24 species) linking range loss to genetic variation loss are predicted well by species *F*_ST_ values. However, middle range values are predicted less well. (b) Single power curve models for the high *F*_ST_ and for the low *F*_ST_ species are well supported, suggesting that the report card might simply use course categories for species that reflect a magnitude of *F*_ST_. Note that, as expected, high *F*_ST_ species will have a more rapid loss of genetic variation as their range extent is lost. (c) The ED method (see Faith et al., 2004) indicates loss of genetic variation for a given species as sites are lost from an environmental space. When losses from environmental space are random, the genetic diversity loss indicated by ED again approximates a power curve (linear when the axes are log‐transformed; shown by the black line). However, ED indicates a range of possible degrees of loss of genetic diversity (shown as the shaded area) depending on the actual pattern of site losses in environmental space.

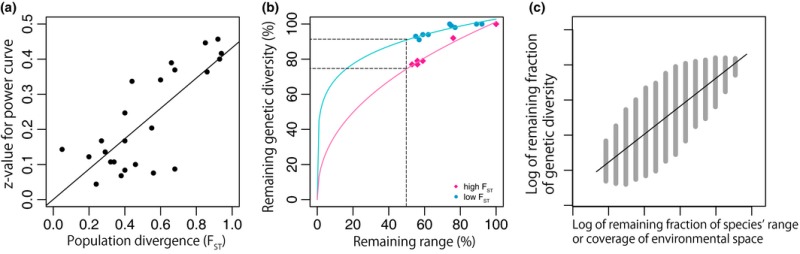

As with direct empirical monitoring, the large subset of specific species for which broad monitoring by proxy would be conducted would need to be selected. If a subset of species is selected that is representative of environmental space, then the estimated loss of genetic diversity for that subset of species may be an indicator of the more general losses. Here, we provide one possible process to select such a set of representative species:

1. For any two species, calculate their “dissimilarity” based on the difference in their locations in environmental (or geographic) space. For example, dissimilarity may be defined as the average distance in environmental space between the locations of the two species.
2. For any nominated target number (call it *k*) of representative species, use the dissimilarities to derive *k* clusters of a species. For example, *k*‐means clustering algorithms can directly use dissimilarities to derive *k* clusters. Choose one member from each cluster to form the subset of *k* representative species.
3. For the *k* species, apply the proxy model to infer loss of genetic variation based on the loss of geographic (or environmental) range extent.

### 4.3 The way forward

A recent study revealed our limited knowledge of global distribution of genetic diversity (Miraldo et al 2016). Thus, we need a global monitoring strategy for intraspecific variation. Such a strategy will require integration and cooperation across researchers, stakeholders, countries, and all levels of government. Many monitoring and database efforts are already underway (e.g., international and national LTER: Long‐Term Ecological Research network; GBIF: Global Biodiversity Information Facility; TRY: plant trait database). For instance, LTER programs collect and archive individual data (e.g., presence/absence, density, and growth data) from multiple plots with repeated observations. Genotyping such individuals contributes to developing global databases of spatiotemporal trends in intraspecific variation. Together with these monitoring efforts, genetic variation can be mapped with other demographic data, including trends in species distribution and population growth. This can be used to account for changes in these trends, to understand how genetic mechanisms are altering population performances, to interpret the effects of the consequences of human activities on ecosystem function via changes in genetic variation, and to illustrate the relative importance of genetic variation among other factors in an ecological and evolutionary context. The discussion that attended such efforts would also serve to predict the changes, identify key gaps in monitoring, assess current indicators and improve them, seek appropriate funding for expanding and better coordinating monitoring efforts, and aid the design and promotion of standardized protocols that could be implemented across taxa and contexts. Settling on these protocols will involve discussion and optimization of answers to the above questions, such as which species to monitor, what types of variation to monitor, and what spatiotemporal scales to monitor. The integrated development and implementation of broad‐brush modeling approaches for situations where direct empirical monitoring is not feasible will also be valuable. To achieve all of these goals, collaboration with other ecological and environmental monitoring networks will be essential.

## 5 Conclusion

The status of, and trends in, intraspecific (genetic and phenotypic) variation through time and space will determine the fate of a population and species, the biodiversity and structure of communities, and the state of ecosystem functions and services. Human activities are having profound effects on variation within and among many populations and species through increased population size, novel selection, and increased gene flow (connections between species/populations), and can thereby shape all of those fates. For these reasons, it is critical to (i) establish monitoring programs for genetic variation, (ii) link observed changes in variation to specific environmental changes and management decisions, and (iii) develop predictive frameworks for changes in variation and its consequences. We call for the development of a globally‐coordinated observation network for monitoring intraspecific variation and its potential consequences for human well‐being.
